# (Nitrato-κ^2^ *O*,*O*′)bis­(tryptanthrin-κ*N*)silver(I)

**DOI:** 10.1107/S1600536812001821

**Published:** 2012-01-21

**Authors:** Jie Wu, Chao Huang, Guo-Qiang Li, Hai-Yan Tian, Ren-Wang Jiang

**Affiliations:** a Guangdong Province Key Laboratory of Pharmacodynamic Constituents of Traditional Chinese Medicine and New Drugs Research, Institute of Traditional Chinese Medicine and Natural Products, Jinan University, Guangzhou 510632, People’s Republic of China; b Sinopharmtcm Shenzhen Ltd, Shenzhen 518029, People’s Republic of China

## Abstract

In the crystal structure of the title compound, [Ag(NO_3_)(C_15_H_8_N_2_O_2_)_2_], tryptanthrin (indolo[2,1-*b*]quinazoline-6,12-dione) and silver nitrate form a 2:1 complex. The silver ion is surrounded by two tryptanthrin ligands, each coordinating through the N atoms, with Ag—N bond lengths of 2.247 (3) and 2.264 (3) Å, and an anionic nitrate ligand coordinating through two O atoms, with Ag—O bond lengths of 2.499 (3) and 2.591 (3) Å. The N—Ag—N plane and the O—Ag—O plane are roughly perpendicular, making a dihedral angle of 81.6 (2)°. In the crystal, C—H⋯O inter­actions between aromatic H atoms and keto and nitrate O atoms as well as π–π inter­actions [centroid-centroid distance = 3.706 (4) Å] give rise to a three-dimensional network.

## Related literature

For the biological activity of tryptanthrin, see: Yu *et al.* (2007 ▶); Chan *et al.* (2009 ▶); Bandekar *et al.* (2010 ▶). For the synthesis and structural modification of tryptanthrin, see: Jao *et al.* (2008 ▶); Kumar *et al.* (2011 ▶); Chen *et al.* (2011 ▶). For related π–π inter­actions in natural flavonoids, see: Jiang *et al.* (2002 ▶, 2009 ▶). For standard bond lengths, see: Allen *et al.* (1987 ▶). For bond lengths and angles in a silver nitrate complex with 4,4′-trimethyl­enedipiperidine, see: Kokunov *et al.* (2011 ▶).
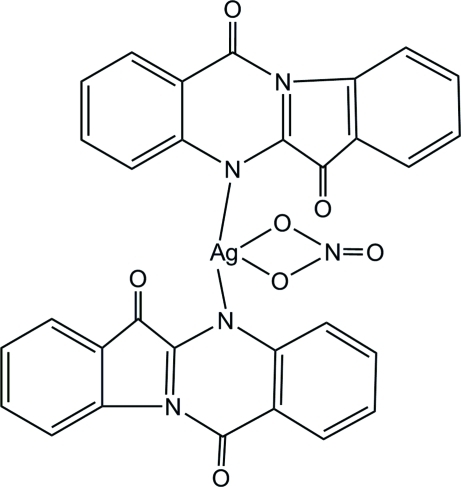


## Experimental

### 

#### Crystal data

[Ag(NO_3_)(C_15_H_8_N_2_O_2_)_2_]
*M* *_r_* = 666.35
Triclinic, 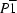

*a* = 8.0598 (19) Å
*b* = 10.873 (3) Å
*c* = 14.541 (3) Å
α = 76.010 (4)°
β = 81.019 (4)°
γ = 84.447 (4)°
*V* = 1219.0 (5) Å^3^
*Z* = 2
Mo *K*α radiation
μ = 0.89 mm^−1^
*T* = 150 K
0.34 × 0.26 × 0.22 mm

#### Data collection

Bruker SMART 1000 CCD diffractometer
Absorption correction: multi-scan (*SADABS*; Sheldrick, 2004 ▶) *T*_min_ = 0.626, *T*_max_ = 1.000
9843 measured reflections
5150 independent reflections
3893 reflections with *I* > 2σ(*I*)
*R*_int_ = 0.034

#### Refinement

*R*[*F* ^2^ > 2σ(*F* ^2^)] = 0.043
*wR*(*F* ^2^) = 0.108
*S* = 1.06
5150 reflections
388 parameters
H-atom parameters constrained
Δρ_max_ = 1.08 e Å^−3^
Δρ_min_ = −0.63 e Å^−3^

### 

Data collection: *SMART* (Bruker, 1998 ▶); cell refinement: *SMART* and *SAINT* (Bruker, 1998 ▶); data reduction: *XPREP* (Bruker, 1998 ▶); program(s) used to solve structure: *SHELXTL* (Sheldrick, 2008 ▶); program(s) used to refine structure: *SHELXTL*; molecular graphics: *XP* in *SHELXTL*; software used to prepare material for publication: *SHELXTL*.

## Supplementary Material

Crystal structure: contains datablock(s) I, global. DOI: 10.1107/S1600536812001821/rn2096sup1.cif

Structure factors: contains datablock(s) I. DOI: 10.1107/S1600536812001821/rn2096Isup2.hkl

Additional supplementary materials:  crystallographic information; 3D view; checkCIF report

## Figures and Tables

**Table 1:** Hydrogen-bond geometry (Å, °)

*D*—H⋯*A*	*D*—H	H⋯*A*	*D*⋯*A*	*D*—H⋯*A*
C3′—H3′*A*⋯O1^i^	0.95	2.52	3.348 (4)	145
C4′—H4′*A*⋯O4^ii^	0.95	2.42	3.300 (3)	153
C3—H3*A*⋯O1′^iii^	0.95	2.45	3.139 (4)	130
C6′—H6′*A*⋯O2	0.95	2.40	3.313 (4)	161
C4—H4*A*⋯O3^iv^	0.95	2.53	3.190 (2)	127
C6—H6*A*⋯O2′	0.95	2.53	3.454 (3)	164
C13′—H13*B*⋯O4^v^	0.95	2.53	3.453 (2)	163
C14—H14*A*⋯O1	0.95	2.47	2.996 (5)	115
C14′—H14*B*⋯O1′	0.95	2.43	2.970 (3)	116

Symmetry codes: (i) 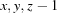; (ii) 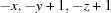; (iii) 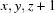; (iv) 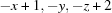; (v) 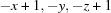.

**Fig. 1.**
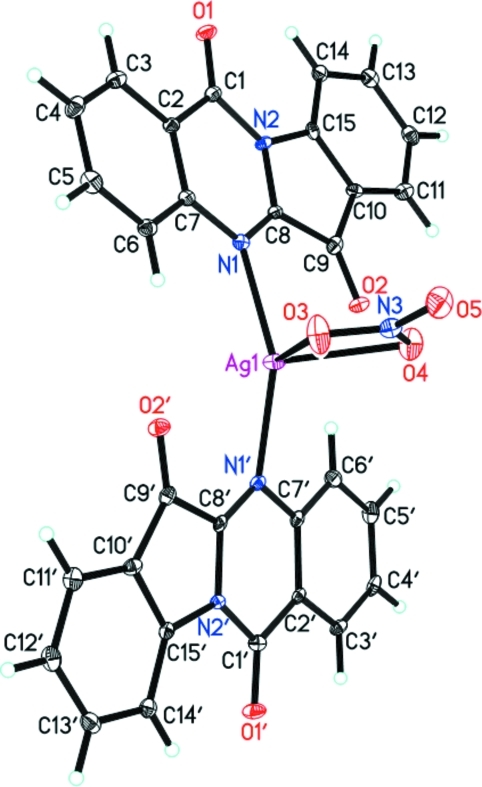
The molecular structure of the title compound showing 30% probability displacement ellipsoids.

**Fig. 2.**
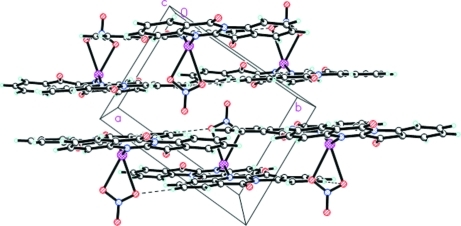
The packing diagram viewed down the c axis. The dashed lines represent intermolecular C—H···O interactions.

## supplementary crystallographic information

### Comment

Tryptanthrin is an indole quinazoline alkaloid isolated from Folium Isatidis (Chen *et al.*, 2011). It was found to inhibit the murine myelomonocytic leukemia cells by causing cell cycle arrest and triggering cell differentiation (Chan *et al.*, 2009), and reverse the doxorubicin resistance in breast cancer cells by inhibition of MDR1 (Yu *et al.*, 2007). It was also found to inhibit the growth of *Escherichia coli* through intercalation into DNA (Bandekar *et al.*, 2010). In addition, synthesis of tryptanthrin (Kumar *et al.*, 2011) and its derivatives (Chen *et al.*, 2011) were reported.

Tryptanthrin and silver nitrate form a 2:1 complex. The same proportions were used in the synthesis. Both the two tryptanthrin molecules are essentially planar with mean deviations of 0.0066 (4) Å and 0.0054 (3) Å, respectively, and make a dihedral angle of 2.9 (2)°. The nitrate group makes dihedral angles of 89.5 (3)° and 105.1 (4)° with the two tryptanthrin molecules. The silver ion is coordinated with two oxygen atoms from the anionic nitrate ligand with bond distances of 2.499 (3) Å and 2.591 (3) Å, and two nitrogen atoms from the tryptanthrin ligands with bond distances of 2.266 (3) Å and 2.249 (3) Å, which are slightly longer than those reported in silver nitrate complex with 4,4'-trimethylenedipiperidine [2.192 (5) Å and 2.212 (5) Å] (Kokunov *et al.*, 2011). The N—Ag—N plane and the O—Ag—O plane are roughly perpendicular with a dihedral angle of 81.6 (2)°. The bond distances and bond angles in both tryptanthrin molecules are all normal (Allen *et al.*, 1987).

Short intermolecular C—H···O interactions (Table 1) between the tryptanthrin methane H atoms and the nitrate ligand [C4'—H···O4, 3.300 (3) Å, C13'—H···O4, 3.453 (2) Å] linked adjacent molecules into layers. Adjacent layers were linked by π-π interactions between the benzene rings [centroid-centroid distance 3.706 (4) Å and displacement angle 4.5 (2)°]. The centroid-centroid distance observed in title compound is similar to those in natural flavonoids (Jiang, *et al.*, 2002 and 2009).

### Experimental

Tryptanthrin (12.4 mg, 0.05 mmol) was dissolved in a solution including methanol (1 ml) and chloroform (3 ml). silver nitrate (17.0 mg, 0.1 mmol) was dissolved in methanol (1 ml) and was added into the tryptanthrin solution. Three days later, the crystals were obtained *via* slow evaporation at room temperature.

### Refinement

The C-bound H atoms were positioned geometrically and were included in the refinement in the riding-model approximation, with C—H = 0.96 Å (CH_3_) and *U*_iso_(H) = 1.5*U*_eq_(C); 0.97 Å (CH_2_) and *U*_iso_(H) = 1.2*U*_eq_(C); 0.93 Å (aryl H) and *U*_iso_(H)= 1.2*U*_eq_(C); O—H = 0.82 Å and *U*_iso_(H) = 1.5*U*_eq_(O). There was a residue peak with height of 1.08 and distance of 0.93 Å from Ag after the final refinement.

### Crystal data

**Table:**

[Ag(NO_3_)(C_15_H_8_N_2_O_2_)_2_]	*Z* = 2
*M**_r_* = 666.35	*F*(000) = 668
Triclinic, *P*1	*D*_x_ = 1.815 Mg m^−^^3^
*a* = 8.0598 (19) Å	Mo *K*α radiation, λ = 0.71074 Å
*b* = 10.873 (3) Å	Cell parameters from 9843 reflections
*c* = 14.541 (3) Å	θ = 3.2–80.9°
α = 76.010 (4)°	µ = 0.89 mm^−^^1^
β = 81.019 (4)°	*T* = 150 K
γ = 84.447 (4)°	Block, yellow
*V* = 1219.0 (5) Å^3^	0.34 × 0.26 × 0.22 mm

### Data collection

**Table:**

Bruker SMART 1000 CCD diffractometer	5150 independent reflections
Radiation source: fine-focus sealed tube	3893 reflections with *I* > 2σ(*I*)
graphite	*R*_int_ = 0.034
ω scans	θ_max_ = 27.1°, θ_min_ = 1.5°
Absorption correction: multi-scan (*SADABS*; Sheldrick, 2004)	*h* = −10→10
*T*_min_ = 0.626, *T*_max_ = 1.000	*k* = −13→13
9843 measured reflections	*l* = −18→18

### Refinement

**Table:**

Refinement on *F*^2^	Primary atom site location: structure-invariant direct methods
Least-squares matrix: full	Secondary atom site location: difference Fourier map
*R*[*F*^2^ > 2σ(*F*^2^)] = 0.043	Hydrogen site location: inferred from neighbouring sites
*wR*(*F*^2^) = 0.108	H-atom parameters constrained
*S* = 1.06	*w* = 1/[σ^2^(*F*_o_^2^) + (0.0565*P*)^2^] where *P* = (*F*_o_^2^ + 2*F*_c_^2^)/3
5150 reflections	(Δ/σ)_max_ = 0.001
388 parameters	Δρ_max_ = 1.08 e Å^−^^3^
0 restraints	Δρ_min_ = −0.63 e Å^−^^3^

### Special details

**Table:**

Geometry. All e.s.d.'s (except the e.s.d. in the dihedral angle between two l.s. planes) are estimated using the full covariance matrix. The cell e.s.d.'s are taken into account individually in the estimation of e.s.d.'s in distances, angles and torsion angles; correlations between e.s.d.'s in cell parameters are only used when they are defined by crystal symmetry. An approximate (isotropic) treatment of cell e.s.d.'s is used for estimating e.s.d.'s involving l.s. planes.
Refinement. Refinement of *F*^2^ against ALL reflections. The weighted *R*-factor *wR* and goodness of fit *S* are based on *F*^2^, conventional *R*-factors *R* are based on *F*, with *F* set to zero for negative *F*^2^. The threshold expression of *F*^2^ > σ(*F*^2^) is used only for calculating *R*-factors(gt) *etc*. and is not relevant to the choice of reflections for refinement. *R*-factors based on *F*^2^ are statistically about twice as large as those based on *F*, and *R*- factors based on ALL data will be even larger.

### Fractional atomic coordinates and isotropic or equivalent isotropic displacement parameters (Å^2^)

**Table:**

	*x*	*y*	*z*	*U*_iso_*/*U*_eq_	
Ag1	0.16812 (4)	0.20968 (3)	0.75957 (2)	0.01945 (11)	
O1	−0.1263 (4)	0.2779 (3)	1.17705 (19)	0.0228 (7)	
O2	−0.0402 (3)	0.4293 (3)	0.76517 (19)	0.0203 (6)	
N1	0.0735 (4)	0.2171 (3)	0.9133 (2)	0.0153 (7)	
N2	−0.1018 (4)	0.3331 (3)	1.0138 (2)	0.0152 (7)	
C1	−0.0672 (5)	0.2537 (4)	1.1009 (3)	0.0181 (9)	
C2	0.0488 (5)	0.1456 (4)	1.0870 (3)	0.0159 (8)	
C3	0.0992 (5)	0.0596 (4)	1.1675 (3)	0.0206 (9)	
H3A	0.0532	0.0691	1.2298	0.025*	
C4	0.2162 (5)	−0.0393 (4)	1.1561 (3)	0.0239 (10)	
H4A	0.2493	−0.0986	1.2108	0.029*	
C5	0.2856 (5)	−0.0522 (4)	1.0649 (3)	0.0229 (9)	
H5A	0.3680	−0.1191	1.0579	0.028*	
C6	0.2361 (5)	0.0311 (4)	0.9844 (3)	0.0196 (9)	
H6A	0.2821	0.0206	0.9223	0.024*	
C7	0.1178 (5)	0.1310 (4)	0.9955 (3)	0.0155 (8)	
C8	−0.0295 (5)	0.3110 (4)	0.9265 (3)	0.0152 (8)	
C9	−0.0852 (5)	0.4188 (4)	0.8498 (3)	0.0181 (9)	
C10	−0.1937 (5)	0.5033 (4)	0.9006 (3)	0.0155 (8)	
C11	−0.2774 (5)	0.6174 (4)	0.8666 (3)	0.0206 (9)	
H11A	−0.2692	0.6537	0.7998	0.025*	
C12	−0.3733 (5)	0.6781 (4)	0.9311 (3)	0.0236 (9)	
H12A	−0.4334	0.7566	0.9089	0.028*	
C13	−0.3827 (5)	0.6249 (4)	1.0287 (3)	0.0215 (9)	
H13A	−0.4495	0.6685	1.0721	0.026*	
C14	−0.2977 (5)	0.5102 (4)	1.0649 (3)	0.0198 (9)	
H14A	−0.3052	0.4746	1.1317	0.024*	
C15	−0.2014 (5)	0.4501 (4)	0.9990 (3)	0.0171 (8)	
O1'	0.1922 (4)	0.0444 (3)	0.37176 (19)	0.0238 (7)	
O2'	0.3255 (4)	−0.0370 (3)	0.76130 (19)	0.0211 (6)	
N1'	0.1282 (4)	0.1466 (3)	0.6288 (2)	0.0151 (7)	
N2'	0.2432 (4)	0.0131 (3)	0.5259 (2)	0.0146 (7)	
C1'	0.1695 (5)	0.0793 (4)	0.4463 (3)	0.0162 (8)	
C2'	0.0650 (5)	0.1903 (4)	0.4645 (3)	0.0147 (8)	
C3'	−0.0166 (5)	0.2676 (4)	0.3917 (3)	0.0168 (8)	
H3'A	−0.0020	0.2486	0.3304	0.020*	
C4'	−0.1183 (5)	0.3713 (4)	0.4083 (3)	0.0189 (9)	
H4'A	−0.1730	0.4244	0.3584	0.023*	
C5'	−0.1411 (5)	0.3985 (4)	0.4989 (3)	0.0223 (9)	
H5'A	−0.2129	0.4695	0.5102	0.027*	
C6'	−0.0611 (5)	0.3240 (4)	0.5718 (3)	0.0202 (9)	
H6'A	−0.0778	0.3431	0.6332	0.024*	
C7'	0.0446 (5)	0.2203 (4)	0.5550 (3)	0.0143 (8)	
C8'	0.2189 (5)	0.0498 (4)	0.6114 (3)	0.0146 (8)	
C9'	0.3156 (5)	−0.0455 (4)	0.6808 (3)	0.0168 (8)	
C10'	0.3883 (5)	−0.1396 (4)	0.6284 (3)	0.0172 (8)	
C11'	0.4838 (5)	−0.2517 (4)	0.6561 (3)	0.0212 (9)	
H11B	0.5066	−0.2801	0.7203	0.025*	
C12'	0.5456 (5)	−0.3218 (4)	0.5889 (3)	0.0234 (9)	
H12B	0.6109	−0.3991	0.6070	0.028*	
C13'	0.5123 (5)	−0.2793 (4)	0.4945 (3)	0.0223 (9)	
H13B	0.5586	−0.3270	0.4488	0.027*	
C14'	0.4125 (5)	−0.1686 (4)	0.4660 (3)	0.0188 (9)	
H14B	0.3877	−0.1409	0.4022	0.023*	
C15'	0.3514 (5)	−0.1012 (4)	0.5342 (3)	0.0152 (8)	
N3	0.4813 (4)	0.3383 (3)	0.7421 (2)	0.0211 (8)	
O3	0.4698 (4)	0.2218 (3)	0.7760 (3)	0.0418 (9)	
O4	0.3608 (4)	0.3988 (3)	0.7064 (2)	0.0355 (8)	
O5	0.6079 (4)	0.3907 (3)	0.7475 (2)	0.0393 (8)	

### Atomic displacement parameters (Å^2^)

**Table:**

	*U*^11^	*U*^22^	*U*^33^	*U*^12^	*U*^13^	*U*^23^
Ag1	0.02012 (18)	0.02486 (19)	0.01477 (17)	0.00208 (12)	−0.00300 (12)	−0.00824 (13)
O1	0.0281 (16)	0.0265 (16)	0.0124 (14)	0.0007 (13)	0.0007 (12)	−0.0053 (12)
O2	0.0196 (15)	0.0273 (16)	0.0136 (14)	−0.0003 (13)	−0.0010 (12)	−0.0053 (12)
N1	0.0112 (16)	0.0187 (17)	0.0167 (17)	−0.0020 (14)	−0.0016 (13)	−0.0051 (14)
N2	0.0131 (16)	0.0203 (17)	0.0115 (16)	−0.0012 (14)	0.0017 (13)	−0.0043 (14)
C1	0.018 (2)	0.022 (2)	0.015 (2)	−0.0074 (17)	−0.0030 (17)	−0.0028 (17)
C2	0.016 (2)	0.018 (2)	0.0147 (19)	−0.0034 (16)	−0.0044 (16)	−0.0036 (16)
C3	0.020 (2)	0.025 (2)	0.016 (2)	−0.0025 (18)	−0.0014 (17)	−0.0045 (18)
C4	0.022 (2)	0.027 (2)	0.021 (2)	−0.0049 (19)	−0.0064 (18)	0.0017 (19)
C5	0.017 (2)	0.022 (2)	0.030 (2)	0.0029 (18)	−0.0062 (18)	−0.0070 (19)
C6	0.018 (2)	0.023 (2)	0.019 (2)	−0.0032 (17)	−0.0033 (17)	−0.0063 (18)
C7	0.0135 (19)	0.018 (2)	0.0160 (19)	−0.0045 (16)	−0.0042 (16)	−0.0035 (16)
C8	0.0145 (19)	0.020 (2)	0.0123 (19)	−0.0090 (17)	−0.0009 (15)	−0.0037 (16)
C9	0.0124 (19)	0.023 (2)	0.018 (2)	−0.0071 (17)	−0.0027 (16)	−0.0007 (17)
C10	0.0113 (19)	0.021 (2)	0.016 (2)	−0.0022 (16)	−0.0004 (16)	−0.0079 (17)
C11	0.016 (2)	0.027 (2)	0.019 (2)	−0.0009 (18)	−0.0040 (17)	−0.0050 (18)
C12	0.022 (2)	0.021 (2)	0.031 (2)	−0.0011 (18)	−0.0100 (19)	−0.0066 (19)
C13	0.014 (2)	0.024 (2)	0.027 (2)	−0.0031 (17)	0.0012 (17)	−0.0087 (19)
C14	0.017 (2)	0.022 (2)	0.020 (2)	−0.0067 (17)	0.0038 (17)	−0.0063 (18)
C15	0.015 (2)	0.0149 (19)	0.022 (2)	−0.0061 (16)	−0.0017 (17)	−0.0039 (17)
O1'	0.0287 (17)	0.0304 (17)	0.0143 (14)	0.0009 (13)	−0.0066 (13)	−0.0076 (13)
O2'	0.0225 (15)	0.0246 (16)	0.0161 (15)	0.0026 (12)	−0.0059 (12)	−0.0041 (12)
N1'	0.0143 (16)	0.0175 (17)	0.0127 (16)	−0.0034 (14)	−0.0008 (13)	−0.0018 (14)
N2'	0.0185 (17)	0.0136 (16)	0.0117 (16)	−0.0010 (14)	−0.0022 (14)	−0.0027 (13)
C1'	0.0128 (19)	0.020 (2)	0.017 (2)	−0.0006 (16)	−0.0020 (16)	−0.0056 (17)
C2'	0.0135 (19)	0.0169 (19)	0.0121 (18)	−0.0051 (16)	−0.0008 (15)	0.0005 (16)
C3'	0.0123 (19)	0.023 (2)	0.0137 (19)	−0.0034 (17)	−0.0002 (16)	−0.0027 (17)
C4'	0.0123 (19)	0.021 (2)	0.019 (2)	−0.0041 (17)	−0.0036 (16)	0.0054 (17)
C5'	0.016 (2)	0.016 (2)	0.031 (2)	0.0045 (17)	−0.0034 (18)	−0.0005 (18)
C6'	0.020 (2)	0.021 (2)	0.021 (2)	−0.0048 (18)	−0.0043 (17)	−0.0047 (18)
C7'	0.0108 (18)	0.0157 (19)	0.0151 (19)	−0.0038 (16)	−0.0007 (15)	−0.0010 (16)
C8'	0.0119 (19)	0.017 (2)	0.0139 (19)	−0.0077 (16)	0.0006 (15)	−0.0003 (16)
C9'	0.0132 (19)	0.018 (2)	0.017 (2)	−0.0021 (16)	0.0000 (16)	−0.0006 (17)
C10'	0.0132 (19)	0.022 (2)	0.0155 (19)	−0.0035 (17)	0.0005 (16)	−0.0037 (17)
C11'	0.016 (2)	0.021 (2)	0.025 (2)	0.0013 (17)	−0.0059 (18)	−0.0017 (18)
C12'	0.019 (2)	0.019 (2)	0.031 (2)	0.0002 (18)	−0.0050 (19)	−0.0039 (19)
C13'	0.019 (2)	0.021 (2)	0.028 (2)	−0.0047 (18)	0.0025 (18)	−0.0094 (19)
C14'	0.016 (2)	0.019 (2)	0.021 (2)	−0.0078 (17)	0.0020 (17)	−0.0041 (17)
C15'	0.0147 (19)	0.0148 (19)	0.0155 (19)	−0.0029 (16)	−0.0036 (16)	−0.0007 (16)
N3	0.0160 (18)	0.026 (2)	0.0206 (19)	0.0022 (16)	−0.0019 (15)	−0.0065 (16)
O3	0.0239 (18)	0.0247 (18)	0.071 (3)	0.0026 (14)	−0.0111 (17)	0.0015 (17)
O4	0.0246 (18)	0.0298 (18)	0.047 (2)	0.0059 (14)	−0.0132 (16)	0.0029 (16)
O5	0.0257 (18)	0.048 (2)	0.045 (2)	−0.0132 (16)	−0.0080 (16)	−0.0051 (17)

### Geometric parameters (Å, °)

**Table:**

Ag1—N1'	2.247 (3)	O1'—C1'	1.215 (5)
Ag1—N1	2.264 (3)	O2'—C9'	1.212 (5)
Ag1—O3	2.499 (3)	N1'—C8'	1.277 (5)
Ag1—O4	2.591 (3)	N1'—C7'	1.398 (5)
O1—C1	1.214 (5)	N2'—C8'	1.374 (5)
O2—C9	1.209 (5)	N2'—C1'	1.393 (5)
N1—C8	1.284 (5)	N2'—C15'	1.439 (5)
N1—C7	1.400 (5)	C1'—C2'	1.457 (5)
N2—C8	1.377 (5)	C2'—C3'	1.392 (5)
N2—C1	1.401 (5)	C2'—C7'	1.413 (5)
N2—C15	1.426 (5)	C3'—C4'	1.376 (6)
C1—C2	1.463 (6)	C3'—H3'A	0.9500
C2—C3	1.398 (5)	C4'—C5'	1.400 (6)
C2—C7	1.399 (5)	C4'—H4'A	0.9500
C3—C4	1.384 (6)	C5'—C6'	1.377 (6)
C3—H3A	0.9500	C5'—H5'A	0.9500
C4—C5	1.390 (6)	C6'—C7'	1.393 (5)
C4—H4A	0.9500	C6'—H6'A	0.9500
C5—C6	1.383 (6)	C8'—C9'	1.511 (5)
C5—H5A	0.9500	C9'—C10'	1.447 (5)
C6—C7	1.397 (6)	C10'—C11'	1.386 (6)
C6—H6A	0.9500	C10'—C15'	1.402 (5)
C8—C9	1.497 (5)	C11'—C12'	1.386 (6)
C9—C10	1.458 (5)	C11'—H11B	0.9500
C10—C11	1.373 (6)	C12'—C13'	1.397 (6)
C10—C15	1.401 (5)	C12'—H12B	0.9500
C11—C12	1.373 (6)	C13'—C14'	1.394 (6)
C11—H11A	0.9500	C13'—H13B	0.9500
C12—C13	1.390 (6)	C14'—C15'	1.375 (5)
C12—H12A	0.9500	C14'—H14B	0.9500
C13—C14	1.390 (6)	N3—O4	1.231 (4)
C13—H13A	0.9500	N3—O5	1.237 (4)
C14—C15	1.385 (6)	N3—O3	1.250 (4)
C14—H14A	0.9500		
			
N1'—Ag1—N1	147.76 (11)	C8'—N1'—C7'	116.9 (3)
N1'—Ag1—O3	114.47 (12)	C8'—N1'—Ag1	116.4 (3)
N1—Ag1—O3	94.10 (12)	C7'—N1'—Ag1	124.8 (2)
N1'—Ag1—O4	108.49 (11)	C8'—N2'—C1'	123.1 (3)
N1—Ag1—O4	101.36 (11)	C8'—N2'—C15'	109.4 (3)
O3—Ag1—O4	49.28 (10)	C1'—N2'—C15'	127.5 (3)
C8—N1—C7	116.5 (3)	O1'—C1'—N2'	121.8 (4)
C8—N1—Ag1	116.7 (3)	O1'—C1'—C2'	125.8 (4)
C7—N1—Ag1	126.7 (2)	N2'—C1'—C2'	112.4 (3)
C8—N2—C1	122.7 (3)	C3'—C2'—C7'	119.5 (4)
C8—N2—C15	109.3 (3)	C3'—C2'—C1'	119.6 (3)
C1—N2—C15	127.9 (3)	C7'—C2'—C1'	120.9 (3)
O1—C1—N2	121.7 (4)	C4'—C3'—C2'	120.2 (4)
O1—C1—C2	126.3 (4)	C4'—C3'—H3'A	119.9
N2—C1—C2	112.0 (3)	C2'—C3'—H3'A	119.9
C3—C2—C7	119.6 (4)	C3'—C4'—C5'	119.9 (4)
C3—C2—C1	118.7 (3)	C3'—C4'—H4'A	120.1
C7—C2—C1	121.6 (3)	C5'—C4'—H4'A	120.1
C4—C3—C2	119.8 (4)	C6'—C5'—C4'	121.0 (4)
C4—C3—H3A	120.1	C6'—C5'—H5'A	119.5
C2—C3—H3A	120.1	C4'—C5'—H5'A	119.5
C3—C4—C5	120.2 (4)	C5'—C6'—C7'	119.4 (4)
C3—C4—H4A	119.9	C5'—C6'—H6'A	120.3
C5—C4—H4A	119.9	C7'—C6'—H6'A	120.3
C6—C5—C4	120.9 (4)	C6'—C7'—N1'	119.0 (3)
C6—C5—H5A	119.6	C6'—C7'—C2'	120.0 (4)
C4—C5—H5A	119.6	N1'—C7'—C2'	121.0 (3)
C5—C6—C7	119.2 (4)	N1'—C8'—N2'	125.6 (4)
C5—C6—H6A	120.4	N1'—C8'—C9'	126.3 (3)
C7—C6—H6A	120.4	N2'—C8'—C9'	108.1 (3)
C6—C7—C2	120.3 (4)	O2'—C9'—C10'	131.1 (4)
C6—C7—N1	118.4 (3)	O2'—C9'—C8'	124.3 (4)
C2—C7—N1	121.2 (3)	C10'—C9'—C8'	104.6 (3)
N1—C8—N2	126.0 (4)	C11'—C10'—C15'	119.7 (4)
N1—C8—C9	125.9 (3)	C11'—C10'—C9'	131.0 (4)
N2—C8—C9	108.1 (3)	C15'—C10'—C9'	109.2 (3)
O2—C9—C10	130.4 (4)	C10'—C11'—C12'	119.0 (4)
O2—C9—C8	124.4 (4)	C10'—C11'—H11B	120.5
C10—C9—C8	105.1 (3)	C12'—C11'—H11B	120.5
C11—C10—C15	121.3 (4)	C11'—C12'—C13'	120.3 (4)
C11—C10—C9	130.6 (4)	C11'—C12'—H12B	119.9
C15—C10—C9	108.1 (3)	C13'—C12'—H12B	119.9
C12—C11—C10	118.6 (4)	C14'—C13'—C12'	121.5 (4)
C12—C11—H11A	120.7	C14'—C13'—H13B	119.2
C10—C11—H11A	120.7	C12'—C13'—H13B	119.2
C11—C12—C13	120.2 (4)	C15'—C14'—C13'	117.2 (4)
C11—C12—H12A	119.9	C15'—C14'—H14B	121.4
C13—C12—H12A	119.9	C13'—C14'—H14B	121.4
C12—C13—C14	122.2 (4)	C14'—C15'—C10'	122.3 (4)
C12—C13—H13A	118.9	C14'—C15'—N2'	129.2 (4)
C14—C13—H13A	118.9	C10'—C15'—N2'	108.5 (3)
C15—C14—C13	116.9 (4)	O4—N3—O5	121.6 (4)
C15—C14—H14A	121.5	O4—N3—O3	117.8 (3)
C13—C14—H14A	121.5	O5—N3—O3	120.6 (4)
C14—C15—C10	120.7 (4)	N3—O3—Ag1	98.2 (2)
C14—C15—N2	129.9 (4)	N3—O4—Ag1	94.3 (2)
C10—C15—N2	109.4 (3)		
			
N1'—Ag1—N1—C8	−87.6 (3)	O4—Ag1—N1'—C7'	−64.6 (3)
O3—Ag1—N1—C8	119.3 (3)	C8'—N2'—C1'—O1'	−179.5 (4)
O4—Ag1—N1—C8	70.0 (3)	C15'—N2'—C1'—O1'	−0.3 (6)
N1'—Ag1—N1—C7	93.5 (3)	C8'—N2'—C1'—C2'	−0.1 (5)
O3—Ag1—N1—C7	−59.6 (3)	C15'—N2'—C1'—C2'	179.1 (3)
O4—Ag1—N1—C7	−108.8 (3)	O1'—C1'—C2'—C3'	−1.2 (6)
C8—N2—C1—O1	176.6 (4)	N2'—C1'—C2'—C3'	179.4 (3)
C15—N2—C1—O1	1.8 (6)	O1'—C1'—C2'—C7'	178.6 (4)
C8—N2—C1—C2	−1.6 (5)	N2'—C1'—C2'—C7'	−0.8 (5)
C15—N2—C1—C2	−176.4 (3)	C7'—C2'—C3'—C4'	−1.0 (6)
O1—C1—C2—C3	0.0 (6)	C1'—C2'—C3'—C4'	178.8 (3)
N2—C1—C2—C3	178.2 (3)	C2'—C3'—C4'—C5'	−0.6 (6)
O1—C1—C2—C7	−175.9 (4)	C3'—C4'—C5'—C6'	1.0 (6)
N2—C1—C2—C7	2.3 (5)	C4'—C5'—C6'—C7'	0.3 (6)
C7—C2—C3—C4	−0.3 (6)	C5'—C6'—C7'—N1'	178.7 (3)
C1—C2—C3—C4	−176.3 (4)	C5'—C6'—C7'—C2'	−1.9 (6)
C2—C3—C4—C5	1.0 (6)	C8'—N1'—C7'—C6'	177.5 (3)
C3—C4—C5—C6	−1.6 (6)	Ag1—N1'—C7'—C6'	−18.6 (5)
C4—C5—C6—C7	1.5 (6)	C8'—N1'—C7'—C2'	−2.0 (5)
C5—C6—C7—C2	−0.8 (6)	Ag1—N1'—C7'—C2'	162.0 (3)
C5—C6—C7—N1	177.8 (3)	C3'—C2'—C7'—C6'	2.3 (5)
C3—C2—C7—C6	0.2 (6)	C1'—C2'—C7'—C6'	−177.5 (3)
C1—C2—C7—C6	176.0 (4)	C3'—C2'—C7'—N1'	−178.3 (3)
C3—C2—C7—N1	−178.4 (3)	C1'—C2'—C7'—N1'	1.9 (5)
C1—C2—C7—N1	−2.5 (6)	C7'—N1'—C8'—N2'	1.0 (5)
C8—N1—C7—C6	−176.8 (3)	Ag1—N1'—C8'—N2'	−164.3 (3)
Ag1—N1—C7—C6	2.1 (5)	C7'—N1'—C8'—C9'	−177.9 (3)
C8—N1—C7—C2	1.8 (5)	Ag1—N1'—C8'—C9'	16.8 (5)
Ag1—N1—C7—C2	−179.3 (3)	C1'—N2'—C8'—N1'	0.0 (6)
C7—N1—C8—N2	−1.2 (5)	C15'—N2'—C8'—N1'	−179.3 (3)
Ag1—N1—C8—N2	179.9 (3)	C1'—N2'—C8'—C9'	179.1 (3)
C7—N1—C8—C9	175.6 (3)	C15'—N2'—C8'—C9'	−0.2 (4)
Ag1—N1—C8—C9	−3.3 (5)	N1'—C8'—C9'—O2'	−4.1 (6)
C1—N2—C8—N1	1.2 (6)	N2'—C8'—C9'—O2'	176.8 (4)
C15—N2—C8—N1	176.9 (4)	N1'—C8'—C9'—C10'	176.7 (4)
C1—N2—C8—C9	−176.0 (3)	N2'—C8'—C9'—C10'	−2.4 (4)
C15—N2—C8—C9	−0.4 (4)	O2'—C9'—C10'—C11'	3.8 (7)
N1—C8—C9—O2	0.9 (6)	C8'—C9'—C10'—C11'	−177.0 (4)
N2—C8—C9—O2	178.2 (4)	O2'—C9'—C10'—C15'	−174.9 (4)
N1—C8—C9—C10	−177.1 (4)	C8'—C9'—C10'—C15'	4.2 (4)
N2—C8—C9—C10	0.2 (4)	C15'—C10'—C11'—C12'	2.0 (6)
O2—C9—C10—C11	1.2 (7)	C9'—C10'—C11'—C12'	−176.6 (4)
C8—C9—C10—C11	178.9 (4)	C10'—C11'—C12'—C13'	0.3 (6)
O2—C9—C10—C15	−177.7 (4)	C11'—C12'—C13'—C14'	−2.1 (6)
C8—C9—C10—C15	0.0 (4)	C12'—C13'—C14'—C15'	1.5 (6)
C15—C10—C11—C12	−1.5 (6)	C13'—C14'—C15'—C10'	0.8 (6)
C9—C10—C11—C12	179.7 (4)	C13'—C14'—C15'—N2'	−178.2 (4)
C10—C11—C12—C13	0.8 (6)	C11'—C10'—C15'—C14'	−2.6 (6)
C11—C12—C13—C14	−0.2 (6)	C9'—C10'—C15'—C14'	176.3 (3)
C12—C13—C14—C15	0.2 (6)	C11'—C10'—C15'—N2'	176.6 (3)
C13—C14—C15—C10	−0.8 (6)	C9'—C10'—C15'—N2'	−4.5 (4)
C13—C14—C15—N2	−179.8 (4)	C8'—N2'—C15'—C14'	−177.9 (4)
C11—C10—C15—C14	1.5 (6)	C1'—N2'—C15'—C14'	2.8 (6)
C9—C10—C15—C14	−179.4 (3)	C8'—N2'—C15'—C10'	2.9 (4)
C11—C10—C15—N2	−179.3 (3)	C1'—N2'—C15'—C10'	−176.3 (3)
C9—C10—C15—N2	−0.3 (4)	O4—N3—O3—Ag1	−7.0 (4)
C8—N2—C15—C14	179.5 (4)	O5—N3—O3—Ag1	170.6 (3)
C1—N2—C15—C14	−5.2 (6)	N1'—Ag1—O3—N3	97.8 (3)
C8—N2—C15—C10	0.4 (4)	N1—Ag1—O3—N3	−97.6 (3)
C1—N2—C15—C10	175.8 (4)	O4—Ag1—O3—N3	3.9 (2)
N1—Ag1—N1'—C8'	−103.8 (3)	O5—N3—O4—Ag1	−170.9 (3)
O3—Ag1—N1'—C8'	46.6 (3)	O3—N3—O4—Ag1	6.7 (4)
O4—Ag1—N1'—C8'	99.4 (3)	N1'—Ag1—O4—N3	−110.7 (2)
N1—Ag1—N1'—C7'	92.3 (3)	N1—Ag1—O4—N3	81.7 (3)
O3—Ag1—N1'—C7'	−117.4 (3)	O3—Ag1—O4—N3	−3.9 (2)

### Hydrogen-bond geometry (Å, °)

**Table:**

*D*—H···*A*	*D*—H	H···*A*	*D*···*A*	*D*—H···*A*
C3'—H3'A···O1^i^	0.95	2.52	3.348 (4)	145
C4'—H4'A···O4^ii^	0.95	2.42	3.300 (3)	153
C3—H3A···O1'^iii^	0.95	2.45	3.139 (4)	130
C6'—H6'A···O2	0.95	2.40	3.313 (4)	161
C4—H4A···O3^iv^	0.95	2.53	3.190 (2)	127
C6—H6A···O2'	0.95	2.53	3.454 (3)	164
C13'—H13B···O4^v^	0.95	2.53	3.453 (2)	163
C14—H14A···O1	0.95	2.47	2.996 (5)	115
C14'—H14B···O1'	0.95	2.43	2.970 (3)	116

Symmetry codes: (i) *x*, *y*, *z*−1; (ii) −*x*, −*y*+1, −*z*+1; (iii) *x*, *y*, *z*+1; (iv) −*x*+1, −*y*, −*z*+2; (v) −*x*+1, −*y*, −*z*+1.
